# Chromosomes trapped in micronuclei are liable to segregation errors

**DOI:** 10.1242/jcs.214742

**Published:** 2018-07-09

**Authors:** Mar Soto, Iraia García-Santisteban, Lenno Krenning, René H. Medema, Jonne A. Raaijmakers

**Affiliations:** Oncode Institute, Division of Cell Biology, The Netherlands Cancer Institute, Plesmanlaan 121, 1066 CX, Amsterdam, The Netherlands

**Keywords:** Micronucleus, Chromosome segregation, Aneuploidy

## Abstract

DNA in micronuclei is likely to get damaged. When shattered DNA from micronuclei gets reincorporated into the primary nucleus, aberrant rearrangements can take place, a phenomenon referred to as chromothripsis. Here, we investigated how chromatids from micronuclei act in subsequent divisions and how this affects their fate. We observed that the majority of chromatids derived from micronuclei fail to establish a proper kinetochore in mitosis, which is associated with problems in chromosome alignment, segregation and spindle assembly checkpoint activation. Remarkably, we found that, upon their formation, micronuclei already display decreased levels of important kinetochore assembly factors. Importantly, these defects favour the exclusion of the micronucleus over the reintegration into the primary nucleus over several divisions. Interestingly, the defects observed in micronuclei are likely overcome once micronuclei are reincorporated into the primary nuclei, as they further propagate normally. We conclude that the formation of a separate small nuclear entity represents a mechanism for the cell to delay the stable propagation of excess chromosome(s) and/or damaged DNA, by inducing kinetochore defects.

## Abstract (precis)

**Highlighted Article:** Micronuclei, which are common in cancer cells, are prone to missegregation owing to kinetochore assembly defects. This represents a mechanism to delay the reincorporation of excess chromosome(s) and/or damaged DNA.

## INTRODUCTION

The presence of micronuclei is a hallmark of chromosome instability. Micronuclei are formed when one or a few chromosomes fail to join a daughter nucleus and form their own nuclear envelope (Crasta et al., 2012). Micronuclei appear to be structurally comparable to primary nuclei, but display reduced functioning in transcription, replication and DNA damage repair (Terradas et al., 2016). These defects are likely a consequence of reduced nuclear pore protein levels in micronuclei leading to impaired micro-nuclear trafficking (Crasta et al., 2012; Hatch et al., 2013; Hoffelder et al., 2004).

During the past years, it has become clear that DNA damage accumulates in micronuclei (Hatch et al., 2013; Zhang et al., 2015). This damage has been suggested to be a starting point for chromothripsis (Zhang et al., 2015), where one or multiple chromosomes acquire dozens to hundreds of clustered rearrangements in a single catastrophic event (Stephens et al., 2011). Chromothripsis is common in cancer and associated with poor prognosis (Rode et al., 2016; Stephens et al., 2011). One of the current models for chromothripsis involves DNA shattering in micronuclei followed by reincorporation into a daughter nucleus, where random religation can take place (Ly et al., 2017). Despite the growing interest in micronuclei, little is known about their fate in subsequent cell divisions, which will be key to understand their contribution to cancer development.

Here, we investigated how chromatids from micronuclei confront subsequent divisions, and how cells can prevent the propagation of such potential harmful structures.

## RESULTS AND DISCUSSION

### Mitotic fidelity of micronucleated cells

An imbalanced karyotype has been shown to increase chromosomal instability (Santaguida and Amon, 2015). However, the contribution of micronuclei was not addressed in that study. Here, we made use of chromosomally stable human RPE-1 cells (retinal pigment epithelial cells) in which micronuclei were induced by the co-inhibition of CENP-E and MPS1 (also known as TTK). A low concentration of CENP-E inhibitor (CENP-Ei) inhibits chromosome congression, causing misalignment of one or few chromosomes. In turn, partial MPS1 inhibition allows for mitotic progression in the presence of misaligned chromosomes, mainly resulting in whole-chromosome missegregations (Soto et al., 2017). To avoid cell cycle arrest (Soto et al., 2017), we either transiently depleted p53 (also known as TP53) with siRNA or used RPE-1 cells harbouring a stable knockdown of p53 (p53kd).

**Fig. 1.**
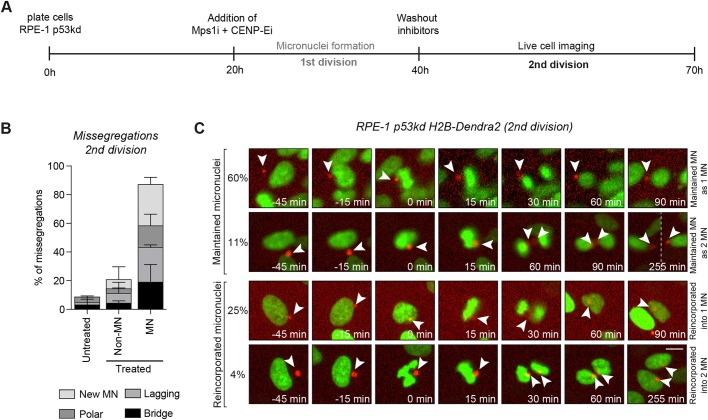
**Chromatids from micronuclei fail to align and are more prone to missegregate.** (A) Experimental setup. Mps1i, MPS1 inhibitor (NMS-P715); CENP-Ei, CENP-E inhibitor (GSK923295). (B) Quantification of missegregations of cells from A. Treated cells were categorized based on the absence (non-MN) or presence of a micronucleus (MN). *n*>50 cells/condition from two independent experiments. Data represent mean+s.d. (C) Stills of RPE-1 p53kd H2B-Dendra2 cells with a photoconverted micronucleus. Cells were treated as in A but after inhibitor washout, micronuclei were photoconverted and traced until the subsequent cell division. *n*=55 cells (three independent experiments). Scale bar: 10 μm.

To test whether our *de novo-*induced micronucleated cells displayed higher amounts of chromosome segregation errors than cells with a single nucleus, we scored segregation errors by live-cell imaging of the mitosis following micronucleus formation (‘2nd division’, see Fig. 1A for experimental setup). As expected, untreated cells displayed few missegregation events; 9.4% of erroneous divisions scored by the presence of lagging chromosomes, anaphase bridges or apparently correct divisions with the appearance of a micronucleus (Fig. 1B) (Soto et al., 2017). Also consistent with previous literature on aneuploid cells, we observed that non-micronucleated cells [the ‘treated population’ of which over 90% is aneuploid (Soto et al., 2017)] displayed a slight increase in segregation errors as compared to untreated cells (21.4% versus 9.4%) (Santaguida et al., 2017; Sheltzer et al., 2011; Zhu et al., 2012). This increase could potentially be explained by the presence of structural imbalances, including acentric DNA fragments formed upon chromosome breakage during the first division in the presence of the drugs (Janssen et al., 2011). Moreover, imbalanced karyotypes have also been shown to induce replication stress and thus promote segregation errors (Passerini et al., 2016).

Surprisingly, the analysis of micronucleated cells showed that 86.9% displayed missegregations (Fig. 1B). This result suggests that, although aneuploidy in the main nucleus is associated with a small increase in segregation errors, the fidelity of chromosome segregation is nearly always affected by the presence of a micronucleus.

To test whether segregation errors are restricted to micronuclei-derived chromosomes, we generated a RPE-1 p53kd cell line stably expressing H2B fused to the photo-switchable fluorophore Dendra2. Importantly, the photo-switching procedure itself did not induce segregation errors (Fig. S1). We specifically photo-switched micronuclei in interphase in order to trace them over subsequent divisions. We found that in 71% of the divisions, micronuclei-derived chromatids missegregated and were maintained as micronuclei in either one or both daughter cells (Fig. 1C, upper panels, 60% and 11%, respectively). In contrast, the other 29% of micronuclei were reincorporated in either one or both daughter cells (25% and 4%, respectively) (Fig. 1C, lower panels). These results were consistent with those in previous publications, where micronuclei were induced by nocodazole washout (Crasta et al., 2012). The limited amount of micronuclei that gave rise to two independent micronuclear structures in the subsequent division can be explained by the fact that chromatids in micronuclei often fail to replicate (Crasta et al., 2012; Okamoto et al., 2012), and thus rarely will two sister chromatids be formed. Furthermore, there is likely a small subset of micronuclei that initially harbour more than one chromosome (Soto et al., 2017). Importantly, independently of the fate of the micronucleus, the vast majority of the photo-switched chromatids (77.3%) misaligned. Moreover, the micronuclei-derived chromosomes that did align were still not equally segregated over the two daughter cells. These results strongly indicate that all micronuclei-entrapped chromatids behave abnormally during chromosome segregation.

### Replication defects do not fully explain the mitotic errors of micronuclei

**Fig. 2.**
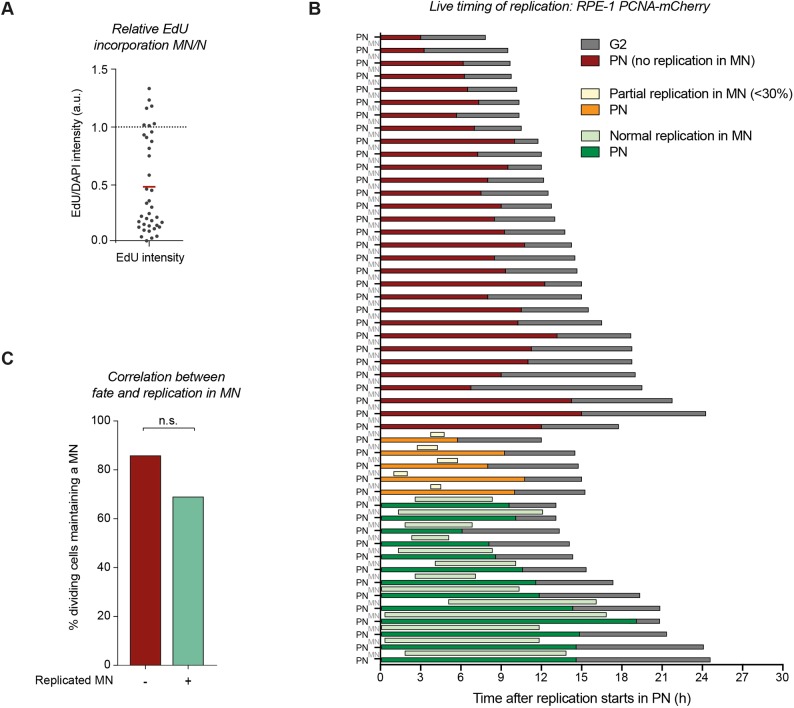
**Replication defects do not fully explain the mitotic errors of micronuclei.** (A) RPE-1 p53kd cells were treated as in Fig. 1A. After washing out the inhibitors, EdU was added for 24 h. The graph depicts the ratio of relative EdU/DAPI levels in the micronucleus (MN) over the primary nucleus (N) (*n*=38 cells from two independent experiments). The red line indicates the mean. a.u., arbitrary units. (B) Micronuclei were induced as in Fig. 1A in p53-depleted RPE-1 cells expressing PCNA–mCherry and H2B–GFP. Replication duration was determined by the appearance and disappearance of PCNA foci in primary nuclei (PN) and respective micronuclei (MN). The end of the bar represents mitotic entry. *n*=49 from three independent experiments. (C) Cells from Fig. 2B plotted depending of the fate of their micronuclei. Chi-squared test: χ^2^=1.815; d.f.=1; *P*=0.1779 (n.s., not significant).

The lack of proper DNA replication in micronuclei could explain their abnormal behaviour. When an unreplicated chromatid participates in mitosis, it can only form monopolar attachments and fails to align properly. In order to test whether replication in micronuclei is impaired, we measured the incorporation of EdU in micronuclei relative to the incorporation of EdU in the main nucleus. A large fraction of micronuclei displayed reduced or no EdU incorporation, indicative of replication failure or replication stress (Crasta et al., 2012; Zhang et al., 2015). However, a significant fraction of cells (∼40%) seemed to display equal EdU levels to those in the primary nucleus (Fig. 2A). To test whether impaired replication could underlie the abnormal behaviour of micronuclei in mitosis, we performed live-cell imaging of micronucleated cells stably expressing fluorescently tagged proliferating cell nuclear antigen (PCNA), and measured the time from the initial appearance to the full disappearance of PCNA foci (representative images shown in Fig. S2) (Bravo and Macdonald-Bravo, 1985; Georgescu et al., 2015; Moldovan et al., 2007). Consistent with the EdU-labelling assay, the majority of micronuclei showed very limited or no replication (Fig. 2B, displayed in orange and red). Also consistent with the EdU-labelling assay, ∼40% of micronuclei displayed near-normal replication timing, comparable to that for their primary nucleus (Fig. 2B). Overall, the timing of replication was usually slightly reduced in micronuclei, which is probably because replication in eukaryotes is characterized by substantial variability in its duration and speed per region (Koren et al., 2014). Thus, replication of the primary nucleus containing (close to) 46 chromosomes is more likely to contain the earliest and the latest outliers than a single chromatid. Most importantly, when we compared the fraction of maintained micronuclei within both populations (replicated and unreplicated), we found that replication status did not significantly determine the fate of micronuclei, as in both categories the majority of micronuclei was maintained (Fig. 2C). However, there seems to be a slight bias for replication-deficient micronuclei to be maintained as micronuclei, but this difference is not statistically significant. We hypothesize that micronuclei that suffer more from replication defects most likely also suffer more from other defects that could interfere with chromosome segregation.

Importantly, the assays that we use to determine replication fidelity are not a full proof that replication is completely accurate. Very minor replication defects could be present even in micronuclei that display full EdU-incorporation and normal PCNA behaviour. Therefore, we cannot exclude that replication defects contribute to the micronuclei-associated segregation errors. However, since we did not find a solid correlation between replication and micronuclear fate, we investigated other possible mechanisms that could explain the abnormal behaviour of micronuclei in mitosis.

### Chromatids from micronuclei fail to activate the SAC and have impaired recruitment of kinetochore proteins

**Fig. 3.**
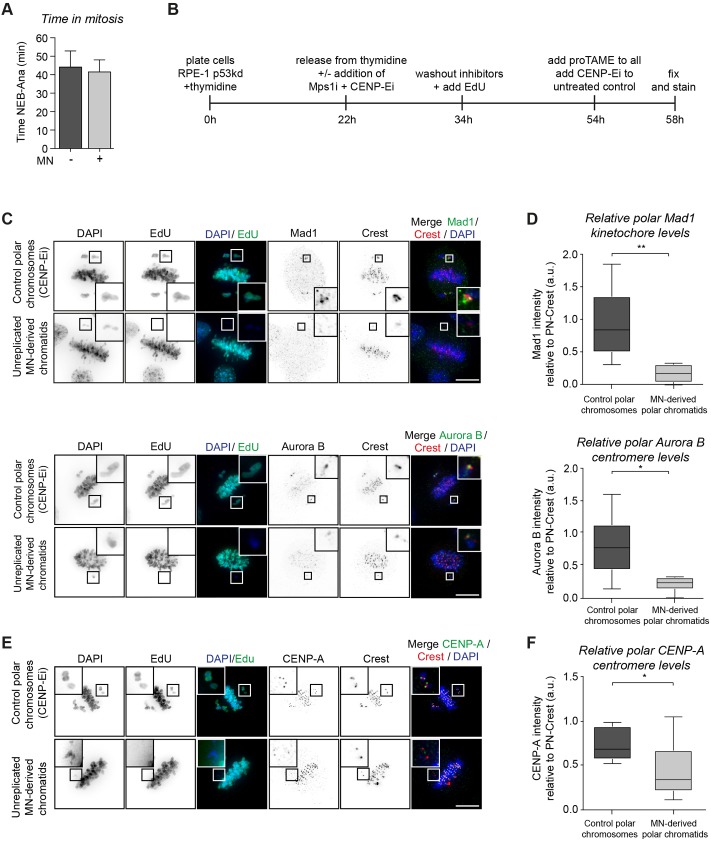
**Chromatids from micronuclei fail to activate the SAC and have impaired recruitment of kinetochore proteins.** (A) Mitotic timing of cells from Fig. 1C. Only cells displaying very clear misalignment of the DNA derived from the micronuclei were analysed (MN+, *n*=17) and compared to untreated cells (MN−, *n*=50). Graph displays mean+s.d. (B) Experimental setup to stain for proteins on micronuclei-derived chromatids. ProTame prevented mitotic exit. Mps1i, MPS1 inhibitor (NMS-P715); CENP-Ei, CENP-E inhibitor (GSK923295). (C) Representative images of RPE-1 p53kd cells displaying reduced levels of Mad1 and Aurora B at micronuclei-derived chromatids. (D) Quantification of fluorescence intensity in cells from C. Data are displayed in box-and-whisker diagrams where the box represents the 25–75th percentiles, and the median is indicated. The whiskers show the range. *n*>6 cells/condition from two independent experiments. (E,F) Same as in C and D but cells were stained for CENP-A. *n*>12 cells/condition from two independent experiments. a.u., arbitrary units. Scale bars: 10 μm.

Under normal conditions, misaligned chromosomes activate the spindle assembly checkpoint (SAC) (Rieder et al., 1994). As shown in Fig. 1A–C, most chromatids derived from micronuclei misalign and missegregate. To test whether micronucleus-derived chromatids were able to trigger a mitotic checkpoint, we determined the time in mitosis of cells from Fig. 1C. Remarkably, cells that misaligned their micronucleus-derived chromatid(s) had a similar mitotic timing to that of untreated cells (Fig. 3A). Checkpoint signalling from only a single chromatid may not be sufficient to induce a mitotic delay (Ibrahim et al., 2017). Alternatively, micronuclei-derived chromatids may have impaired ability to recruit mitotic checkpoint proteins. To test this, we stained for the mitotic checkpoint protein Mad1 (also known as MAD1L1) and for Aurora B, a kinase involved in microtubule attachment and mitotic checkpoint signalling. We used the APC/C inhibitor ProTAME to capture cells in their second division (Zeng et al., 2010) (see Fig. 3B for experimental setup). To identify micronuclei-derived chromatids, we selected chromatids that were misaligning and were EdU-negative, as chromatids in micronuclei often fail to replicate. As a control, we used non-micronucleated cells treated with CENP-Ei to generate polar chromosomes (Fig. 3B). Interestingly, Mad1 levels were severely reduced at EdU-negative chromatids, as compared to polar chromosomes in control cells (Fig. 3C,D, upper panels). Similar results were found for Aurora B (Fig. 3C,D, lower panels). The failure to properly recruit Mad1 and Aurora B potentially reflects a failure to build a functional kinetochore. To test this, we determined the levels of CENP-A, a centromeric-specific variant of histone H3 that plays a key role in kinetochore assembly (Régnier et al., 2005). Consistent with the above hypothesis, CENP-A levels were significantly reduced at micronucleus-derived chromatids (Fig. 3E,F). Thus, the aberrant behaviour of micronuclear-derived chromatids in mitosis is likely a consequence of defective kinetochore assembly, resulting in a failure to attach to the mitotic spindle and to activate the SAC.

### Defects in early interphase lead to impaired kinetochore assembly

**Fig. 4.**
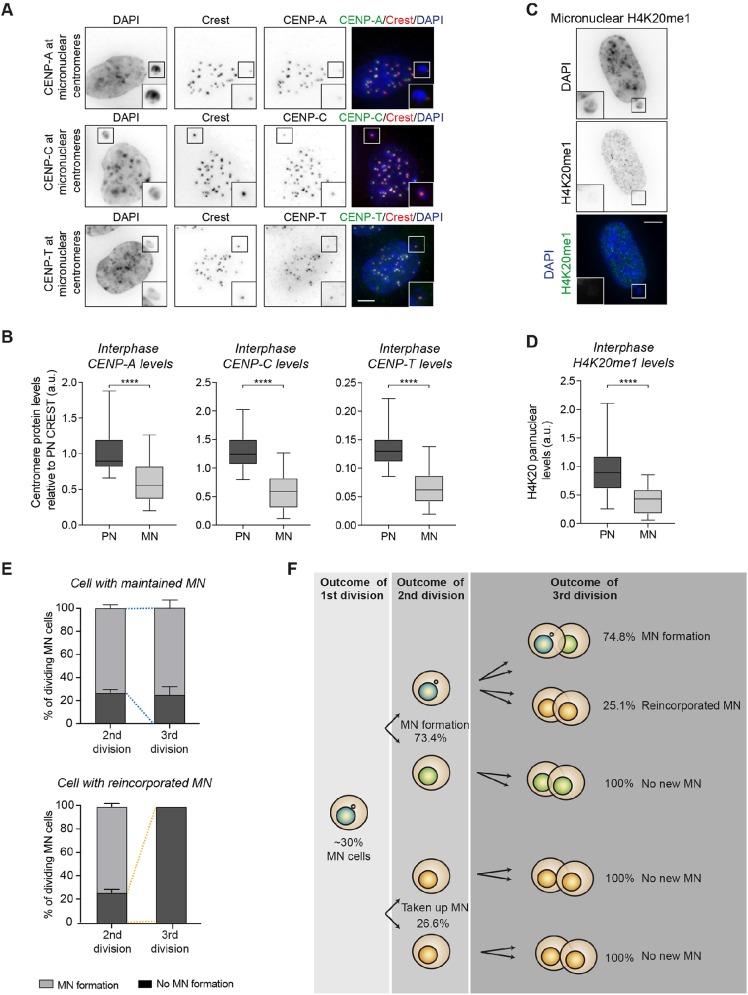
**Kinetochore defects delay micronuclei reincorporation.** (A) Representative images of micronucleated RPE-1 p53kd cells stained for CENP-A, CENP-C and CENP-T. (B) Quantification of fluorescence intensity in cells from A. *n*>22 cells/condition from two independent experiments. (C) Representative images of RPE-1 p53kd cells containing micronuclei stained for H4K20me1. (D) Quantification of fluorescence intensity in cells from C. *n*>27 cells/condition from 2 independent experiments. Data in B and D are displayed in box-and-whisker diagrams where the box represents the 25–75th percentiles, and the median is indicated. The whiskers show the range. MN, micronuclei; N, primary nuclei. (E) Fate of micronuclei in the third division. A distinction was made between cells that maintained their micronucleus (upper graph) or reincorporated the micronucleus (lower graph) into primary nuclei in the second division. Data represent mean±s.d. (F) Representation of the fate of micronuclei over consecutive divisions. Fractions were calculated based on three independent experiments where 61 cells were scored at the second division, and 25 daughters at the third division. a.u., arbitrary units. Scale bars: 5 μm.

CENP-A spreads over the two sister chromatids during replication and is reloaded in the following G1 (Jansen et al., 2007). Hence, we questioned whether CENP-A reloading in micronuclei was impaired in G1. Indeed, CENP-A levels were also reduced in micronuclei of interphase cells (10 h after thymidine release) (Fig. 4A, upper panel; Fig. 4B, left panel). Importantly, it was reported that a failure to load CENP-A during one cell cycle is not sufficient to compromise the functionality of a kinetochore (Hoffmann et al., 2016; Régnier et al., 2005). We therefore determined the levels of other critical proteins for kinetochore assembly: CENP-C and CENP-T (Gascoigne and Cheeseman, 2011). Consistent with the above result, CENP-T and CENP-C levels in micronuclei were also remarkably reduced (Fig. 4A,B). To test whether replication status influences the kinetochore assembly defects, we evaluated CENP-A, CENP-C, CENP-T and CREST levels in both replicated and unreplicated micronuclei in interphase. Interestingly, we observed a ∼50% reduction of all tested protein levels at centromeres in micronuclei, irrespective of their replication status (Fig. S3A). These data indicate that the kinetochore assembly defects are a consequence of very early defects in micronuclei that are already established in G1 and are independent of replication defects.

We also found that H4K20me1, an epigenetic modification essential for kinetochore assembly (Hori et al., 2014), was significantly decreased in micronuclei (Fig. 4C,D). Interestingly, the H4K20me1 methylase (Set8, also known as KMT5A), the chaperone that is responsible for CENP-A loading (HJURP), and as well as CENP-C and CENP-T must undergo active transport via the nuclear pore complexes (Görlich and Kutay, 1999; Stewart, 2007). Interestingly, micronuclei have protein import defects (Crasta et al., 2012; Hatch et al., 2013) owing to a reduced density of nuclear pore complexes (Hoffelder et al., 2004; Terradas et al., 2016). We could confirm a strong import defect in micronuclei in our setup by the use of a synthetic fluorescent reporter of JNK kinase activity based on nuclear translocation (Regot et al., 2014) (Fig. S3B).These results show that kinetochore assembly is severely impaired in micronuclei, probably resulting from a general import defect that is already present in early interphase.

### Kinetochore defects delay micronuclei reincorporation

In order to determine the effect of the described impairments on micronuclear fate over several divisions, we performed long-term live-cell imaging. As expected from Fig. 1C, ∼70% of the divisions led to the maintenance of micronuclei and in the remaining ∼30%, micronuclei were reincorporated. Interestingly, cells that reincorporated the micronucleus did not exhibit new micronucleus formation in the subsequent division, suggesting that the kinetochore impairments in micronuclei are likely overcome when reincorporated (Fig. 4E). Most strikingly, micronucleated cells showed a very consistent outcome over subsequent divisions; ∼75% maintained and ∼25% reincorporated the micronucleus (compare Fig. 1C and Fig. 4F). This suggests a stochastic distribution favouring the exclusion of a micronucleus over the reintegration into the primary nucleus (Fig. 4E). Importantly, sisters of micronucleated cells never formed new micronuclei suggesting that micronucleus formation is not due to chromosome instability of the cells, but is intrinsic to micronuclei-derived chromatids (Fig. 4F).

Taken together, our data describe how chromatids trapped in micronuclei fail to build proper kinetochores and are prone to missegregate over subsequent divisions. Our results are in line with a previous observation in mouse embryos that described repeated micronuclei inheritance and impaired CREST levels (Vázquez-Diez et al., 2016). Importantly, the chromosome segregation errors linked to micronuclei described here could not be linked to replication impairments in micronuclei, but are rather a consequence of kinetochore assembly impairments. We hypothesize that these defects are a consequence of impaired micronuclear import. If true, understanding why distinct processes are differentially affected by import defects needs further investigation since replication impairments are also thought to be a consequence of import defects (Crasta et al., 2012). Interestingly, the kinetochore assembly defects are already present in G1, suggesting that chromatids that end up in micronuclei are liable to defective segregation from the moment of their exclusion from the primary nucleus.

The conclusions presented here provide important insights into the fate of micronuclei in subsequent cell divisions. Importantly, micronuclei have previously been shown to be major players of chromothripsis, and their potential oncogenic impact relies on their reincorporation into the primary nucleus (Crasta et al., 2012; Ly et al., 2017; Zhang et al., 2015). We suggest that such unilateral inheritance and maintained exclusion from the primary nucleus protects cells from chromothripsis-like rearrangements. Further research on the exact role of the aberrant chromothriptic chromosome structures in tumour formation and progression will be key to comprehending the ultimate consequences of chromosomes entrapped in micronuclei.

## MATERIALS AND METHODS

### Cell culture, cell lines and reagents

To construct cell lines stably expressing H2B-Dendra2, HEK293T cells were transfected with LV.CNV.puro.H2B-Dendra2 construct (a gift from Jacco van Rheenen, Molecular Pathology, The Netherlands Cancer Institute, The Netherlands) using X-tremeGENE (Roche) according to manufacturer's protocol. After 2 days, virus-containing medium was added to RPE-1 p53kd cells (a gift from Johan Kuiken and Roderick Beijersbergen, Department of Cellular and Molecular Medicine, The Netherlands Cancer Institute), and Dendra2-positive cells were sorted on green fluorescence at 2 weeks post-infection. RPE-1 cells expressing PCNA–mCherry and H2B–eGFP were kindly provided by Arshad Desai (Ludwig Institute for Cancer Research, USA). To make RPE-1 JNK-KTR cells, HEK293T cells were transfected with pLenti PGK Puro DEST JNKKTRClover (Addgene plasmid #59151, deposited by Markus Covert; Regot et al., 2014) using X-tremeGENE (Roche) according to manufacturer's protocol. The obtained virus was added to RPE-1 and drug selection was performed (puromycin 1 μg/μl) at 24 h post-infection. All cells described above were cultured at 37°C at 5% CO_2_ in advanced Dulbecco's modified Eagle's medium with nutrient mixture F-12 (DMEM-F12) with Glutamax (GIBCO), supplemented with 10% fetal calf serum (Clontech), 100 U/ml penicillin (Invitrogen), 100 μg/ml streptomycin (Invitrogen) and 2 mM UltraGlutamin (Lonza). For cell cycle synchronization, cells were treated with 2.5 mM thymidine (Sigma) for 22 h and released by washing twice with phosphate-buffered saline (PBS). Inhibitors were all dissolved in DMSO and were used at the following concentrations: proTAME, 20 μM; GSK923295, 50 nM; NMS-P715, 480 nM; Anisomicyn, 50 μM (1 h); and JNK inhibitor VIII, 10 μM. All cell lines described above have been shown to be free of mycoplasma contamination.

### Time-lapse imaging

For live-cell imaging, cells were grown in Lab-Tek II chambered coverglass (Thermo Science). Images were acquired every 5, 10 or 15 min using a DeltaVision Elite (Applied Precision) microscope maintained at 37°C, 5% CO_2_ using a 20×0.75 NA lens (Olympus) and a Coolsnap HQ2 camera (Photometrics) with 2 times binning. Image analysis was performed with ImageJ software. For micronuclei tracking experiments, pre-converted micronuclei were identified by green fluorescence and photoconverted by using a brief (0.05 s) pulse of a 405 nm laser on a Deltavision Elite microscope equipped with a X4 laser module (Applied Precision). Subsequent live-cell imaging was performed as stated above. A Lionheart FX automated microscope was used for nuclear import assays (microscope maintained at 37°C, 5% CO_2_ using a 20× NA lens and a Sony CCD, 1.25 megapixel camera with 2 times binning; BioTek).

### siRNA transfection

ON-TARGETplus SMARTpool siRNA targeting p53 (Thermo Scientific) was transfected using RNAiMAX (Life Technologies) according to the manufacturer's protocol at a final concentration of 20 nM, 24 h before the start of the experiment.

### Immunofluorescence

Cells were grown on 10-mm glass coverslips and fixed in 3.7% formaldehyde with 0.5% Triton X-100 in PBS for 15 min at room temperature. Primary antibodies were incubated at 4°C overnight and secondary antibodies were incubated for 2 h at room temperature, both dissolved in PBS 0.1% Tween. The following antibodies were used: Mad1 (1:500, sc-65494, Santa Cruz Biotechnology), Crest (1:5000, CS1058, Cortex Biochem), CENP-A (1:300, ab13939, Abcam), CENP-C (1:600, PD030, MBL), CENP-T (1:1000, D286-3, MBL), H4K20me1 (1:2000, Hori et al., 2014). Secondary antibodies conjugated to Alexa Fluor 488, Alexa Fluor 568 and Alexa Fluor 647 (Molecular Probes) were used for immunofluorescence. DAPI was added to all samples before mounting using Vectashield mounting fluid (Vector Laboratories). Replication levels were determined for cells cultured in medium containing EdU for the indicated time. After fixation, EdU incorporation was visualized by staining with buffer (100 mM Tris-HCl pH 8.5, with 1 mM CuSO_4_) and Alexa Fluor 488–azide (Life Technologies) according to the manufacturer's protocol. Images were acquired on a DeltaVision Elite microscope (Applied Precision), taking 200-nm *z*-stacks with a PlanApo N 60× NA 1.42 objective (Olympus) and a Coolsnap HQ2 camera (Photometrics). Images were analysed after deconvolution using SoftWoRx (Applied Precision). Figures are maximum intensity projections of entire cells. Brightness and contrast were adjusted with Photoshop 6.0 (Adobe). For kinetochore stainings, since all proteins tested seemed to have lower levels in micronuclei, including centromeric proteins, all measurements were normalized to the average of 10 centromeres (CREST) in the primary nucleus (Fig. 4A,C).

## Supplementary Material

Supplementary information
