# Supplementary material for: Chromosomes trapped in micronuclei are liable to segregation errors
Source: J Cell Sci. 2018 Jul 9;131(13):jcs214742. doi: 10.1242/jcs.214742 (PMC6051344; doi:10.1242/jcs.214742)
Supplement: Supplementary information [file joces-131-214742-s1.pdf]

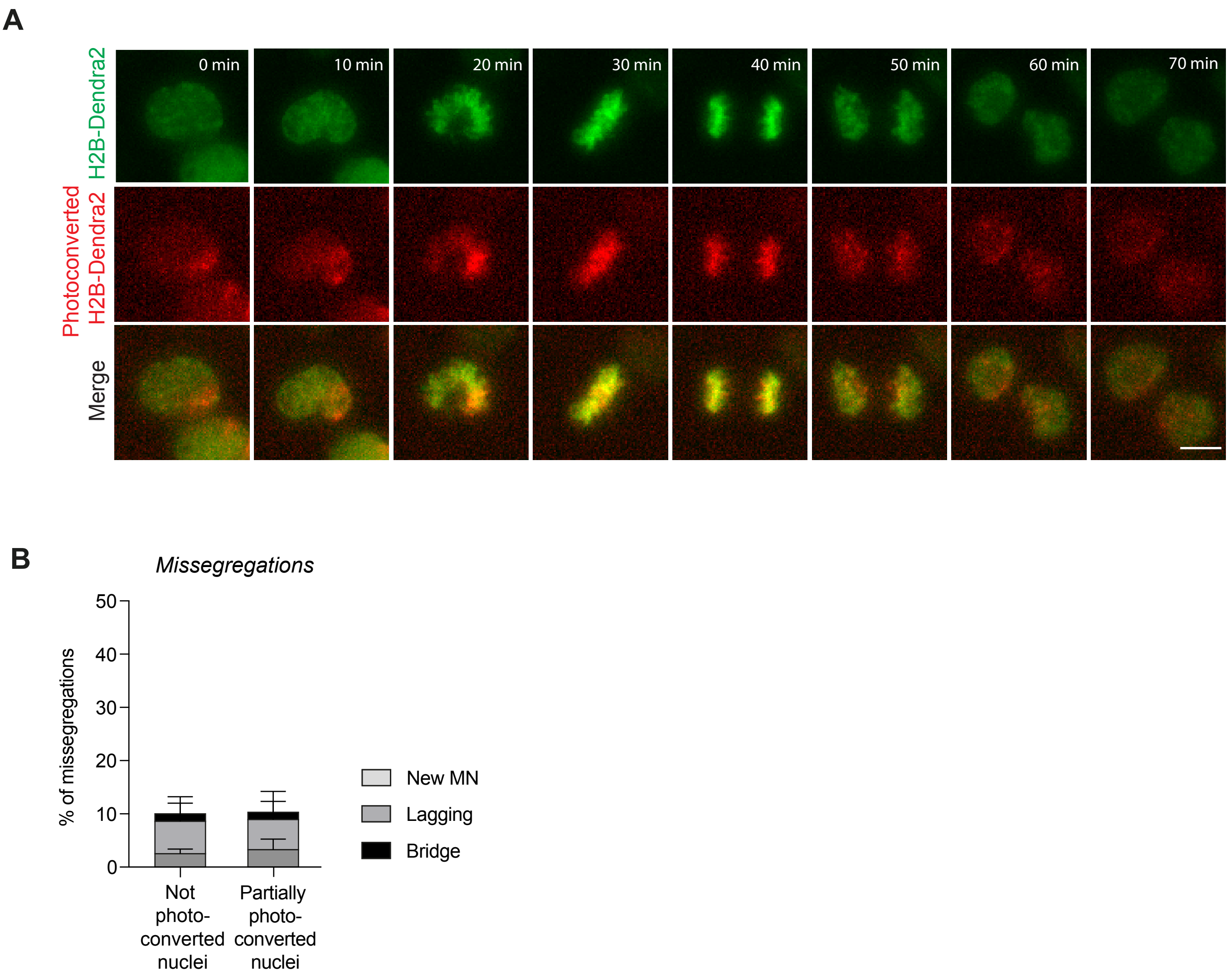

Figure S1. H2B-Dendra2 Photoconversion does not induce chromosome missegregations
A) Example of cell division traced by live cell imaging after photoconverting a fraction of H2B-Dendra2. Scale bar: 5 μm. B) Quantification of missegregation of either untreated control cells (intact nuclei) or cells that that have undergone laser photoconversion in about 50% of the interphase nuclei (photoconverted nuclei). Fractions were calculated based on 2 independent experiments where n1>60 cells per condition and n2>100 per condition. Data are presented as mean + standard deviation.

Journal of Cell Science • Supplementary information

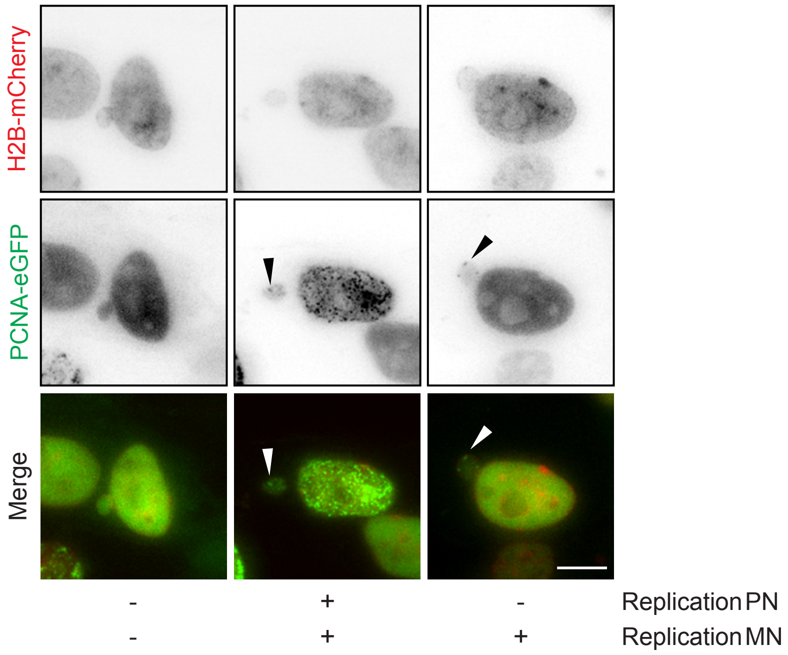

Figure S2. Proliferating cell nuclear antigen (PCNA) foci used to detect replication in nuclei and micronuclei
Representative images of a cell before replication, during replication in the primary nucleus (PN) and the micronucleus (MN), and with only active replication in MN. Scale bar: 5 μm

Journal of Cell Science • Supplementary information

J. Cell Sci. 131: doi:10.1242/jcs.214742: Supplementary information

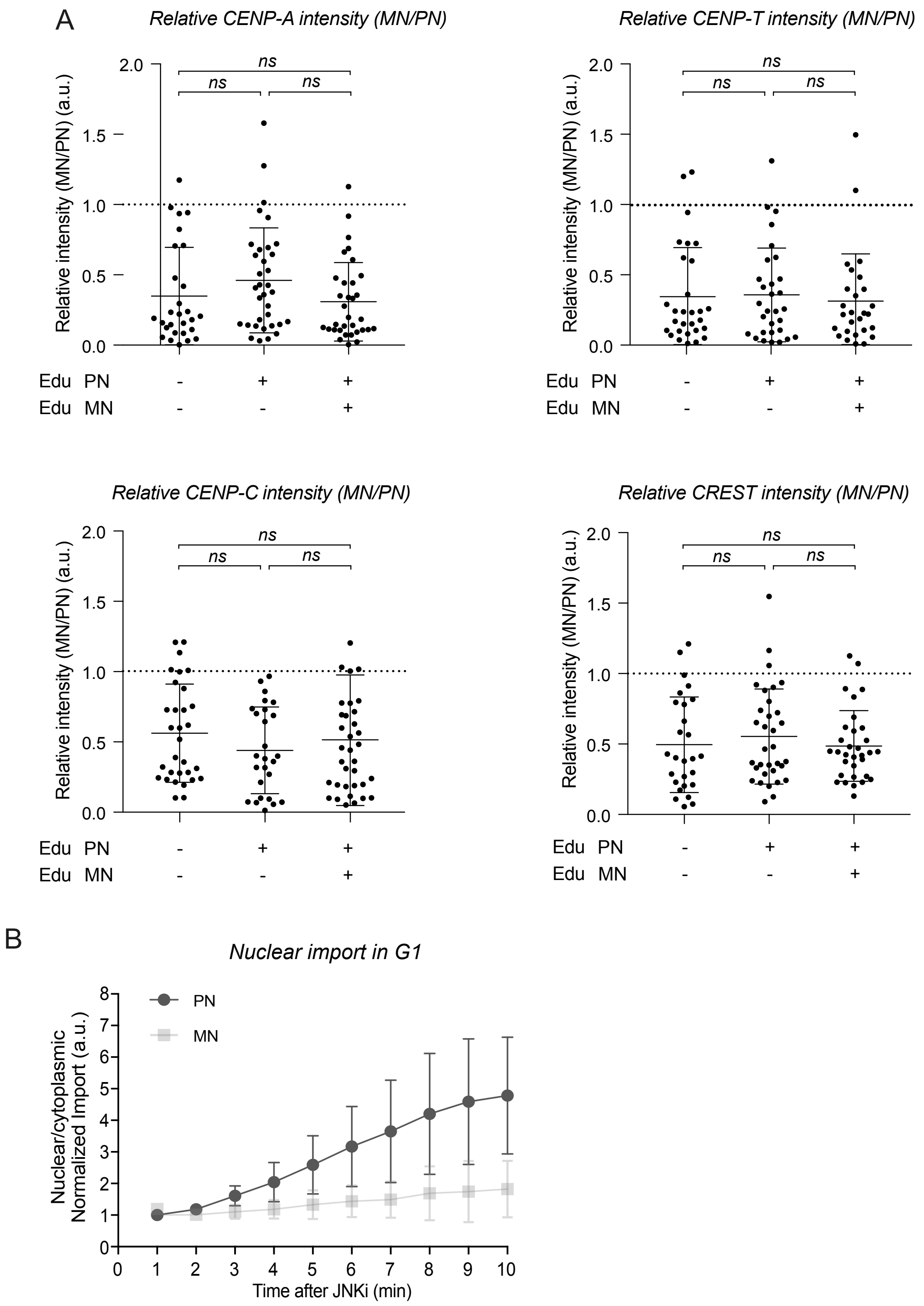

Figure S3. Centromere protein levels throughout the cell cycle and import impairments in micronuclei
A) Quantification of the relative CENP-A, CENP-T, CENP-C and CREST levels in micronuclei (MN)/primary nucleus (PN). Cells have been categorized based on the presence (+) or absence (-) of EdU in MN and PN, as a marker for replication. For each cell, the centromere in the MN was identified by the presence of CREST and divided by the average of 10 kinetochores in the PN. Fractions were calculated based on 2 independent experiments. At least 28 cells were analysed per condition, from 2 independent experiments. Data are displayed in scatter plots displaying individual measurements, means and standard deviations. B) Nuclear and micronuclear import assay as described by Regot and colleagues. The reporter is cytoplasmic when using a JNK activator (anysomicin) and translocates into the nucleus when JNK is inhibited. We induced micronuclei formation in RPE-1 JNK-KTR cells as described in Fig. 3B, but instead of EdU, JNK activator was added to the cells for 1 hour until the totality of the probe was cytoplasmic in all cells. We started live cell imaging of these cells at the same time of the addition of JNK inhibitor. We calculated the nuclear import by dividing the probe intensity in the nucleus or micronucleus over the intensity in the cytoplasm. Data were normalized to the values obtained in the first time point and are presented as average +/- s.d of 10 micronucleated cells.

Journal of Cell Science • Supplementary information
